# Ultra-Trace Analysis of Cyanotoxins by Liquid Chromatography Coupled to High-Resolution Mass Spectrometry

**DOI:** 10.3390/toxins12040247

**Published:** 2020-04-11

**Authors:** Daria Filatova, Oscar Núñez, Marinella Farré

**Affiliations:** 1 Department of Environmental Chemistry, IDAEA-CSIC, 08034 Barcelona, Spain; daria.filatova@idaea.csic.es; 2 Department of Chemical Engineering and Analytical Chemistry, University of Barcelona, 08028 Barcelona, Spain; oscar.nunez@ub.edu; 3 Serra Húnter Professor, Generalitat de Catalunya, 08007 Barcelona, Spain

**Keywords:** Cyanotoxins, surface water, high-resolution mass spectrometry

## Abstract

The increasing frequency of episodes of harmful algal blooms of cyanobacterial origin is a risk to ecosystems and human health. The main human hazard may arise from drinking water supply and recreational water use. For this reason, efficient multiclass analytical methods are needed to assess the level of cyanotoxins in water reservoirs and tackle these problems. This work describes the development of a fast, sensitive, and robust analytical method for multiclass cyanotoxins determination based on dual solid-phase extraction (SPE) procedure using a polymeric cartridge, Oasis HLB (Waters Corporation, Milford, MA, USA), and a graphitized non-porous carbon cartridge, Supelclean^TM^ ENVI-Carb^TM^ (Sigma-Aldrich, St. Louis, MO, USA), followed by ultra-high-performance liquid chromatography high-resolution mass spectrometry (SPE-UHPLC-HRMS). This method enabled the analysis of cylindrospermopsin, anatoxin-a, nodularin, and seven microcystins (MC-LR, MC-RR, MC-YR, MC-LA, MC-LY, MC-LW, MC-LF). The method limits of detection (MLOD) of the validated approach were between 4 and 150 pg/L. The analytical method was applied to assess the presence of the selected toxins in 21 samples collected in three natural water reservoirs in the Ter River in Catalonia (NE of Spain) used to produce drinking water for Barcelona city (Spain).

## 1. Introduction

Cyanobacteria are a group of prokaryotic and photosynthetic organisms that are widespread in freshwater and marine environments. In particular conditions, about 40 different genera [1] can produce secondary metabolites called cyanotoxins to (apparently) defend their living space against other organisms.

Cyanotoxins vary in structure and toxicity, and they may be found within bacterial cells or released into the water. Among them, microcystins (MCs) and nodularins (NODs) are cyclic peptides with hepatotoxic activity containing β-amino acid ADDA (3-amino-9-methoxy-2,6,8-trimethyl-10-phenyldeca-4(E),6(E)-dienoic acid). The common structure of MCs is cyclo(D-Ala-L-**X**-D-erythro methylAsp(iso-linkage)-L-**Z**-Adda-D-Glu(iso-linkage)-N-methyldehydro-Ala). The prime structural difference lies within the L-amino-acid residues 2 (**X**) and 4 (**Z**), which are represented by a two-letter suffix. For instance, MC-LR contains leucine (L) in position 2 and arginine (R) in position 4 [1,2]. Thus, MC-YR is for tyrosine and arginine; -RR is for two arginines; and -LA, -LF, -LY, and -LW are for leucine and alanine, phenylalanine, tyrosine, and tryptophan, respectively. Cylindrospermopsin (CYN) is an alkaloid that has been demonstrated to be hepatotoxic, cytotoxic, dermatotoxic, and possibly carcinogenic [1,3]. Finally, anatoxin-a (ANA) is a bicyclic secondary amine with neurotoxic effects [1,4].

Cyanotoxins can produce adverse effects on human health through drinking [2,5,6] and bathing in contaminated water [7,8]. Some acute events through inhalation have been as well reported, which suggests their potential pass at the airborne phase [8]. Moreover, fish can be exposed to these toxins in the environment and accumulate them in different organs. For example, cylindrospermopsin can be accumulated in fish and cooking processes could degrade the molecule and generate decomposition products [9]. The risk associated with human health, in particular by drinking contaminated water, promoted different regulations to protect consumers. The World Health Organization (WHO) appointed a guideline value of 1 μg/L in drinking water for total MC-LR. This value is provisional since it covers only MC-LR, while reliable toxicological data for other MCs are still unavailable [10]. Moreover, the US National Center for Environmental Assessment suggested lowering the drinking water guideline value to 0.1 μg/L [11]. Effective risk assessment and human health protection require sensitive and efficient detection of a wider spectrum of toxins and their congeners. In this sense, analytical methods producing raw data that can be posteriorly reanalyzed to check for a major number of compounds and metabolites, like those produced by full scan at high-mass resolution, are highly required.

Over the last few years, increasing eutrophication processes and climate change have led to the proliferation, frequency, and persistence of cyanobacteria blooms producing cyanotoxins [12,13]. To assess cyanotoxins varieties, high-throughput analytical approaches for the quantification of multi-class toxins in environmental matrices are required.

Today, the most common analytical techniques for the determination of cyanotoxins are immunological techniques, such as enzyme-linked immunosorbent assay (ELISA), biochemical approaches, and liquid chromatography coupled to mass spectrometric analyzers [14]. High and ultra-high performance liquid chromatography coupled with mass spectrometry (HPLC-MS, UHPLC-MS) and tandem mass spectrometry (MS/MS) are the techniques of choice, in particular with triple quadrupole (QqQ) analyzers [11,15,16,17,18]. However, due to the advances in identification and sensitivity, several HRMS methods have been applied for the determination of cyclic peptides [19,20,21] and multi-class cyanotoxins [22,23,24,25] in freshwater. These methods present limits of detection from 0.3 ng/L to 3900 ng/L, being 0.3–5.6 ng/L the lowest ones reported by Greer et al. [18] by UHPLC-MS/MS.

The goal of the present work was to develop and validate a sensitive and robust analytical method for the determination of cyanotoxins of different chemical classes, such as seven MCs (MC-LR, -RR, -YR, -LA, -LY, -LW, -LF), ANA, CYN, and NOD in water. The proposed method was based on a dual solid-phase extraction (SPE) approach followed by ultra-high-performance liquid chromatography high-resolution mass spectrometry (UHPLC-HRMS). The newly developed approach presented extremely low limits of detection and the advantages of a high degree of confidence in the identification of targeted compounds due to high-mass resolution. The new method was used to investigate the targeted toxins in drinking water reservoirs that are used to provide tap water for Barcelona city (Spain).

## 2. Results and Discussion

### 2.1. SPE Procedure Optimization

Based on previously reported protocols, three cartridges were preselected and tested to achieve effective extraction of targeted cyanotoxins: Oasis HLB (500 mg, 6cc, Waters Corporation, Milford, MA, USA), Supelclean^TM^ ENVI-Carb^TM^ (500 mg, 6cc, Supelco, Sigma-Aldrich, St. Louis, MO, USA), and ISOLUTE^®^ ENV+ (500 mg, 6cc, Biotage, Uppsala, Sweden). For optimization, artificial freshwater (AFW) fortified with 75 ng/L of MC-LY, -LW, and -LF and 100 ng/L for the rest of the selected toxins was used. Concentrations for three MCs (MC-LY, -LW, and -LF) are different due to prime concentration of each standard provided by the producer, which was 7.5 µg/mL for these three cyanotoxins.

As expected, a single cartridge cannot successfully retain all of ten selected cyanotoxins, since they were of different groups and with different physicochemical properties. It was found that Oasis HLB retained MCs and NOD effectively. In contrast, Supelclean^TM^ ENVI-Carb^TM^ was more effective for the retention of CYN and ANA, which is consistent with previously reported studies [26,27,28]. Loading was carried out at neutral pH for the optimization, and the response of the solvent, temperature, and pH for the elution step were studied. In Table 1, the recoveries at the different conditions are presented. As can be seen, MCs were the most efficiently eluted from Oasis HLB with either 10 mL of MeOH at room temperature (25 °C) or 5 mL MeOH heated at 50 °C. Thus, further elution with 10 mL of hot MeOH resulted in 1–9% better recoveries for cyanotoxins. However, MC-LW and MC-LF were slightly better eluted from Oasis HLB with basified MeOH. While CYN was better eluted from Supelclean^TM^ ENVI-Carb^TM^ with acidified MeOH. At this step of optimization, the highest recoveries for ANA were achieved with Oasis HLB with MeOH with formic acid (FA).

To improve the recoveries from the Supelclean^TM^ ENVI-Carb^TM^ cartridge for both CYN and ANA, loading and elution steps were optimized. The mass balance experiment for Supelclean^TM^ ENVI-Carb^TM^ was performed: 250 mL of water at neutral pH spiked with 1 µg/L of ANA and CYN were passed through the cartridge. Water was collected and analyzed. The results obtained showed that ANA was poorly retained, while CYN was retained entirely in Supelclean^TM^ ENVI-Carb^TM^ cartridges at neutral pH (data not shown). In order to improve retention of ANA in the Supelclean^TM^ ENVI-Carb^TM^ cartridge, optimization of loading step was carried out. Different pH (neutral, with 0.1% and 0.01% of ammonium hydroxide (NH_4_OH)) of loading were tested. Back-flush elution with 10 mL of hot MeOH with 0.5% of FA was applied. Back-flush was used for better elution of CYN since it was highly retained in the cartridge. The results obtained showed improvement of CYN and ANA recoveries of up to 68% and 46% respectively, with basified (0.1% of NH_4_OH) loading. Loading with 0.01% of NH_4_OH and neutral pH recovered 60% and 51% of CYN, and 40% and 2% of ANA, respectively.

Finally, it was also observed that better recoveries for all targeted mycotoxins were achieved by increasing the amount of elution solvent up to 20 mL of heated MeOH for Oasis HLB and 20 mL of heated MeOH with 0.5% of FA for Supelclean^TM^ ENVI-Carb^TM^. This allowed to recover up to 2.87% more for cyanotoxins: 2.4% for CYN, 2.65%—ANA, 1.75%—MC-RR, 2.87%—MC-YR, 0.56%—MC-LR, 0.72%—MC-LA, 0.91%—MC-LF. The recoveries for each cartridge are shown in Table S1 (Supplementary Materials), and the final recoveries of the proposed SPE method are summarized in Table 2. 

### 2.2. Liquid Chromatography Coupled to High-Resolution Mass Spectrometry

As a first step, a C_18_ reversed-phase HPLC column (LichtoCART^®^) was employed to optimize the separation conditions of the 10 selected cyanotoxins. MeOH and ACN acidified with FA have been used in general as organic phases in most of the analytical methods previously reported [29] (especially ACN). In Table S2 of the Supplementary Materials, several chromatographic parameters were compared. The tailing factor for ANA and MC-RR was lower with ACN, while for NOD, MC-YR, MC-LR was lower with MeOH. However, when ACN was employed, resolution for NOD, MC-LA, and MC-LW was better. Therefore, ACN was selected as the organic component of the mobile phase. Then, the amount of FA employed in the mobile phase, between 0.05% and 1%, was evaluated. In Figure S1 of the Supplementary Materials, the normalized signal for the studied mycotoxins using the different FA contents is shown. As can be seen, using 0.05% of FA for most of the compounds the highest signal intensities were obtained, except MC-RR and MC-LA. Thus, 0.05% FA was selected as optimum for the determination of the targeted cyanotoxins.

**Figure 1:**
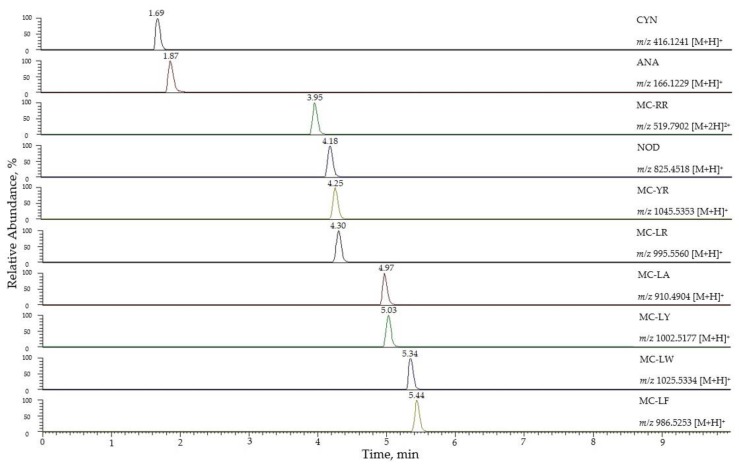
Extracted ion chromatograms for the 10 targeted cyanotoxins at 5 µg/L.

Then, the method was transferred to a C_18_ reversed-phase UHPLC column (Hibar^®^, Merck, Darmstadt, Germany) to reduce the run time. Table S3 (Supplementary Materials) shows the comparison of several chromatographic parameters (retention time, retention factor, asymmetry factor, tailing factor, selectivity, resolution, and peak width) for both columns (HPLC and UHPLC). In general, a better resolution was obtained using the UHPLC column, as expected, except for the pair MC-LW and MC-LF that was better resolved by HPLC. The total analysis time was decreased from 25 min to 10 min when UHPLC was used. Therefore, UHPLC was selected for the optimal method. In Figure 1, the extracted ion chromatograms for the ten targeted cyanotoxins at a concentration of 5 µg/L are shown.

To determine the optimal MS conditions, toxin standards were directly infused into the HESI source. The obtained mass of the corresponding ion was compared to the theoretical mass that was calculated by Xcalibur 2.1 software (Thermo Fisher Scientific, San Jose, CA, USA), for each analyte. Mass deviations expressed in parts per million (ppm), were found to be below 2 ppm (except for CYN and MC-LF in negative ionization mode, which were −2.17 ppm and −2.03 ppm, respectively). As a precondition and precautionary measure for the analysis, the elimination of systematic mass drift by using internal lock mass for each compound was set up. Real-time recalibration on the “lock mass” by correction of shifts helps to remove errors associated with calibration of mass scale.

For further optimization, the ionization of the targeted toxins was studied by flow injection analysis (FIA) using isocratic mobile phase composed by water and ACN, both acidified with 0.1% FA, (50/50, *v*/*v*) at a flow rate of 0.07 mL/min. The concentrations of injected standards were 0.75 mg/L for MC-LY, MC-LW, MC-LF, and 1 mg/mL for the other seven toxins. Capillary temperature (275 °C, 325 °C, 375 °C), heater temperature (225 °C, 275 °C, 325 °C), which were changed pairwise with differences of 50 °C, spray voltage (3 kV, 3.5 KV, 4 kV), and S-lens RF levels (60% and 70%) were evaluated. These tests were performed in both positive and negative ionization modes.

The optimal parameters for both modes were as follows: sheath gas, 10 a.u.; sweep gas, 0 a.u.; auxiliary gas, 5 a.u.; capillary temperature, 320 °C; HESI-II probe temperature, 275 °C; electrospray voltage, 3.5 kV; S-lens RF level, 60%. The selected ionization mode was positive because ANA was not detected in negative mode, as previously reported [30]. In Table S4 (Supplementary Materials), the mass spectral characterization of selected toxins for both ionization modes, as well as the mass deviations, are presented.

During changing from HPLC to UHPLC column ionization parameters were readjusted as the flow was increased from 0.2 mL/min to 0.3 mL/min.

Once full scan HRMS acquisition conditions were established, in order to achieve additional identification points, product ion fragmentation studies were performed. First, the collision energy for each toxin was established. For that purpose, CE was studied from 10 to 60 eV with an increase of 5 eV. CE was optimized more precisely (changing only +/− 2 eV around the chosen value) for the selection of the optimal product ions for quantitation and confirmation purposes. Three parameters were considered: t_R_, and abundances of both product and precursor ions. For the optimization of fragmentation, parallel reaction monitoring mode (PRM) was applied, as compounds were studied separately. PRM mode is normally used for short inclusion lists, as scan speed is not high enough for larger inclusion lists in the same time window. For instance, Roy-Lachapelle et al. applied PRM mode to obtain the MS/MS spectra of five cyanotoxins [24]. To have enough scans per peak, ddMS^2^ and data-independent acquisitions (DIA) could be applied. For ddMS^2^, isolation of precursor ions from the inclusion list or certain intensity trigger fragmentation (if “pick others” parameter in dd Settings is on) was employed. DIA provides fragmentation of all ions or ions in a certain mass range, regardless of the inclusion list. DIA is useful for non-targeted screening or suspect screening with a long list of suspects. DIA mode was recently applied by Roy-Lachapelle et al. [25] for the determination of microcystins. In our case, we focused on 10 commonly found cyanotoxins. Additionally, we tried both switched on and off “pick others parameter” (data not shown). Piking others could provide fragmentation of potential suspects for posterior screening with sufficient signal intensity, however, this provided a lower number of scans per peak of targeted compounds when employing a UHPLC column. Thus, the use of “pick others” was not considered. For future perspectives in posterior suspect screening, full scan in high-resolution and retention time prediction may be applied.

In Table 3, the optimal collision energy values for each toxin, as well as the precursor and product ions selected are summarized. Observed fragmentation is in accordance with previously-reported works in the literature [30,31,32,33,34,35,36,37,38,39,40,41].

The most abundant ions in full scan HRMS mode were chosen for quantification purposes. In the case of MCs, both single [M + H]^+^ and double-charged [M + 2H]^2+^ ions were produced. Arginine-containing MC-RR is known to produce double-charged ions, as the guanidine group in the arginine (Arg) residue is a preferred ionization site. MC-RR contains two arginine residues. Thus, it forms double charged ions easier, and its abundance was significantly higher than that of single charged ions [34,42]. Similarly to MC-RR, MC-LR and MC-YR had also double charged ions with higher abundances. MCs without Arg were protonated at the methoxy group of the ADDA residue forming a singly charged ion. The main transition of both [M + H]^+^ and [M + 2H]^2+^ MCs was either to PhCH_2_CH(OCH_3_) or with the loss of PhCH_2_CH(OCH_2_). The ion at *m/z* 135 is a fragment from the α-cleavage of the methoxy group of the ADDA residue, in agreement with previously reported studies [34,42]. NOD formed the precursor ion [M + H]^+^ because of the protonation of Arg, and the most abundant fragment is again *m/z* 135 due to the protonation of the methoxy group of the ADDA residue [37]. The precursor ion for CYN was [M + H]^+^ at *m/z* 825. The most intense fragment was *m/z* 336 due to the loss of SO_3_ [31]. ANA with [M + H]^+^ precursor ion at *m/z* 166 provided the most abundant product ion at *m/z* 149, corresponding to the loss of the amine NH_3_ [30].

### 2.3. Method Validation and QA/QC

To determine the instrumental limits of detection (ILODs), a standard solution containing the 10 selected cyanotoxins was prepared at an initial concentration of 50 µg/L. The ILODs were determined by progressive dilution with an injection volume of 20 µL. ILODs ranged between 0.02 pg and 1.5 pg on the column (Table 1). On the other hand, ILOQ was calculated as three times the ILOD. In the same way, the method limit of detection (MLOD) of each analyte was defined as the lowest concentration for which the peak area was detected, while method limit of quantification (MLOQ) was established as the relative standard deviation of three replicates, below 19%; Gaussian peak shapes; less than 3 ppm of exact mass error; and molecular isotopic pattern accomplishing the standard ratio. MLODs and MLOQs for the selected toxins were found to be between 4–150 pg/L and 12–450 pg/L, respectively. To our knowledge, these are the lowest reported MLODs for the determination of multi-class cyanotoxins [11,15,16,17,18,22,23,28,43].

Linearity was evaluated by analyzing mixtures of the 10 targeted cyanotoxins at 16 different concentrations in the range 1–50 µg/L, obtaining good linearities with linear regression coefficients (R^2^) below 0.9928 (Figure S2).

Recoveries were estimated by analysis of enriched AFW samples in triplicate with the ten selected cyanotoxins at three concentration levels (1.5 ng/L, 7.5 ng/L, and 15 ng/L for MC-LY, -LW and -LF, and at 2 ng/L, 10 ng/L, and 20 ng/L for the other targeted toxins). Blank matrix samples were treated with the same extraction procedure and then spiked at the same concentration to be used as a reference. The recovery for each toxin was calculated dividing the integrated area obtained for each sample into one of the references for the respective matrix and concentration and then multiplied per 100 to have the % value. The mean recoveries at the lowest, medium, and highest concentration levels were between 53.4–84.3%, 52.2–73.6%, and 66.6–87.3%, respectively, for most of the toxins except for MC-LW that were, in general, at a lower percentage in agreement with the results obtained by other authors [28].

Intra-day and inter-day precision expressed as % RSD was evaluated by repeated replicate determinations of a standard solution as indicated in Section 2.4. Intra-day precision ranged from 2.0% to 8.8%, and inter-day ranged from 2.0% to 23.2% for all toxins, which are acceptable values considering the analytes, matrices, and analytical techniques used.

The matrix effect was also evaluated in AFW enriched in selected toxins within the range between 0.0002 and 0.1 µg/L. Matrix effects, enhancement and inhibition, were observed (Table 1) but did not change significantly within the range of concentration. To solve the matrix effects, matrix-matched calibration curves were used for the quantification of selected cyanotoxins in real samples.

### 2.4. Investigation of Cyanotoxins in Barcelona Water Reservoirs at the Ter River

The applicability and good performance of the proposed analytical approach for the determination of the targeted cyanotoxins were evaluated by analyzing real freshwater samples. For that purpose, a total of twenty-one freshwater samples were collected between March and September 2018 from three different water reservoirs located at the Ter River, in central Catalonia (NE Spain). Samples were processed in triplicate as described in Section 4.3 and analyzed with the developed UHPLC-HRMS method.

**Figure 2:**
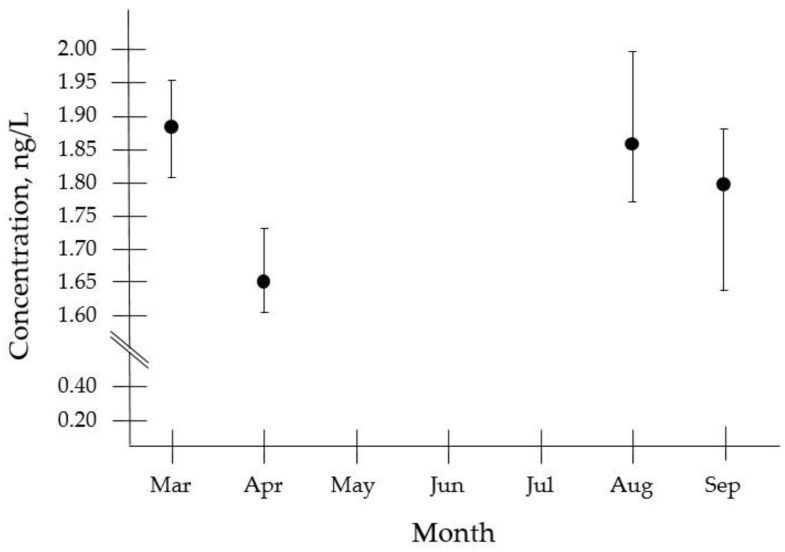
Levels of MC-RR in Susqueda water reservoir. Analyzed in triplicate by the developed method.

The presence of the targeted mycotoxins in the analyzed samples was not relevant. Among the 10 toxins under study, only MC-RR was detected and quantified, and only in 22% of the freshwater samples analyzed. Although detected in the three studied water reservoirs, MC-RR was most frequently found in the Susqueda water reservoir. Figure 2 shows the concentration levels found for MC-RR in the Susqueda water reservoir from March to September 2018. As can be seen, MC-RR was quantified at concentrations within 1.2 ng/L and 1.4 ng/L. Besides, the two peaking months were March and August.

Regarding the other two water reservoirs under study, MC-RR was also found in the Pasteral water reservoir in April and the Sau water reservoir in September, and its concentration was 1.2 ng/L and 1 ng/L, respectively.

Therefore, the proposed method can be applied for the analysis of real water samples achieving the WHO guided value for MC-LR in drinking water.

## 3. Conclusions

A multi-residue method has been developed and evaluated for the analysis of 10 cyanotoxins in freshwater, showing a solid performance at the part-per-trillion level. Sample clean-up and pre-concentration were achieved by applying a two steps SPE protocol with HLB Oasis and Supelclean^TM^ ENVI-Carb^TM^ SPE cartridges. The determination of the targeted compounds was performed using UHPLC-HRMS/MS.

The method was assessed concerning accuracy, specificity, selectivity, repeatability, within-laboratory reproducibility, limits of detection and quantification and linearity. The developed method can be proposed for both environmental and food analysis due to the number of confirmation criteria such as HRMS, and MS/MS ions. Data acquired in full scan can be used for posterior suspect screening of other natural toxins and cyanotoxins.

The capabilities and the good performance of this method were confirmed by analysis of real spiked samples and real freshwater samples from a sampling campaign in the Ter River. The method was applied to characterize the occurrence of these contaminants in samples from the Barcelona water reservoirs located at the Ter River during the months prior and posterior of the seasonal algal blooms. In this case, only MC-RR was detected in less than 25% of the samples. This result is in agreement with the climatic conditions of the investigated year. The year 2018 was especially rainy and with temperatures colder than usual in the NE of Spain.

## 4. Materials and Methods

### 4.1. Chemicals and Reagents

CYN, ANA, MC-LR, MC-RR, MC-YR, MC-LY, MC-LW, and MC-LF (99%) standards were purchased from Cyano Biotech GmbH (Berlin, Germany). NOD and MC-LA were purchased from ENZO life Science (Lausen, Switzerland). Methanol (MeOH), acetonitrile (ACN), and water HPLC grade were obtained from Fisher Scientific (Leics, UK). FA (98%) was purchased from Fluka (Steinheim, Germany). NH_4_OH (25%) was obtained from Sigma-Aldrich (Steinheim, Germany). Sodium chloride, potassium chloride (99.5%), and sodium carbonate (99.9%) were purchased from Merck (Darmstadt, Germany). Calcium chloride (93%), magnesium chloride (98%), and HEPES (99.5%) were obtained from Sigma-Aldrich (Steinheim, Germany). AFW was prepared according to Lipschitz et al. [44], and Na_2_CO_3_ 1M was used for AFW pH adjustment

### 4.2. Samples and Sampling Sites

Twenty-seven freshwater samples were collected from three water reservoirs located at the Ter River, in central Catalonia (NE Spain). Pasteral (41.983040; 2.599138) has a storage capacity of 233 hm^3^. The Susqueda reservoir (41.970002; 2.524971) is located in Osor with a storage capacity of 216 hm³ while the main water body is within the boundaries of Susqueda and Sant Hilari Sacalm. The dam in Sau (41.975693; 2.395398) created a reservoir with a storage capacity of 153.05 hm^3^ that covered the former town of Sant Romà de Sau.

Samples were collected between March and September 2018 covering the months before the expected algal bloom, and July and August, which are the months with maximum insolation and the conditions favoring the potential blooms. The samples were collected in amber glass bottles, and the pH, temperature, pO_2_, and conductivity were measured on-site. Samples were transported at 4 °C and then were frozen at −40 °C until the initiation of the analytical process.

Additionally, surface water samples were spiked at three concentration levels (20 ng/L, 50 ng/L, and 100 ng/L) to demonstrate the applicability of the method for all the determined toxins.

### 4.3. Samples Pretreatment

First, 300 mL of each freshwater sample was ultrasonicated for 30 min at a power of 200 W and a frequency of 60 Hz to disrupt cells and release the intracellular toxins. Then, the samples were centrifuged for 7 min at 3219.84 g. After this process, 250 mL of the supernatant was collected, and the cyanotoxins were isolated by a sequentially solid-phase extraction (SPE) procedure using the following two cartridges: Oasis HLB (500 mg, 6cc, Waters Corporation, Milford, MA, USA) and Supelclean^TM^ ENVI-Carb^TM^ (500 mg, 6cc, Supelco, Sigma-Aldrich, St. Louis, MO, USA). The Oasis cartridges were used in the first step after conditioning with 10 mL of methanol, followed by 10 mL of artificial freshwater. Then, 250 mL of the supernatant of each sample was loaded at 1 mL/min, and the elution was accomplished with 20 mL of MeOH heated at 50 °C.

The percolated sample is then collected, basified up to 0.1% ammonia, and then transferred to the second SPE step with a Supelclean^TM^ ENVI-Carb^TM^ cartridge conditioned with 10 mL of methanol and 10 mL of AFW containing 0.1% NH_4_OH. The elution of the second cartridges was carried out by back-flush elution with 20 mL of MeOH heated at 50 °C containing 0.5% of FA. Both Oasis HLB and Supelclean^TM^ ENVI-Carb^TM^ extracts were combined, dried under a gentle stream of nitrogen, and re-dissolved with 500 µL acetonitrile (ACN):H_2_0 (10:90, *v*/*v*). All the samples were analyzed in triplicates.

### 4.4. Analysis by Liquid Chromatography Coupled with High-Resolution Mass Spectrometry

The analysis was performed using an Accela LC instrument (Thermo Fisher Scientific, San Jose, CA, USA), coupled to a Q-Exactive Orbitrap high-resolution mass spectrometry analyzer (Thermo-Fisher Scientific) equipped with a heated electrospray ionization (HESI) source operating in positive mode. Two chromatographic separations were optimized, using HPLC and UHPLC columns. The first one was achieved using a Lichrosphere C18 reversed-phase column (125 mm × 2 mm i.d., 5 μm) (Merck, Barcelona, ES, Spain) with a mobile phase composed of water (solvent A) and acetonitrile (solvent B) both acidified with 0.1% of FA. The optimal elution gradient was as follows: from 0–3 min, 10% B; from 3–13 min, B was linearly increased to 90%; 13–15 min, stabilized at 90% B; 15–16 min, B decreased linearly to 10%; 16–20 min, column stabilization with a 10% of solvent B. This method was then transferred to a C_18_ UHPLC column Hibar^®^ (2.1 mm × 150 mm, 2 µm particle size) to reduce the run time. The elution gradient was modified accordingly and the optimal separation was achieved using the following one: from 0–1 min at 10% B, from 1–5 min, gradient was linearly increased from 10 to 90% B; from 5–8 min, gradient was linearly decreased at 10% B; from 8–10 min, the column was re-equilibrated at 10% B. The injection volume was 20 µL, and the flow rate was 0.3 mL/min.

The optimal source HESI parameters were set as follows: spray voltage of +4 kV, sheath gas, auxiliary gas and sweep gas at 35, 17, and 1 a.u. (arbitrary units), respectively, the heater temperature at 300 °C, the capillary temperature at 350 °C, and S-lens RF level at 60%. The acquisition was performed in full-scan mode at a resolving power of 70,000 full width at half maximum (FWHM) (*m/z* 200), and the data-dependent MS^2^ (ddMS^2^) mode was acquired at a resolving power of 17,500 FWHM. Collision energy (CE) was optimized for each compound. The full-scan was used for quantification, and the most abundant fragment from ddMS^2^ mode was chosen for confirmation. In Table 3, the selected precursor ions for each cyanotoxin, the obtained product ions, the optimal collision energies, and the tentative ion assignments are summarized.

The positive identification of target toxins was carried out by comparing the retention times of analytes in the samples and standards in matrix-matched AFW with a maximum tolerance of ± 2%. The exact mass tolerance was set at ± 5 ppm for the extracted *m/z* values from acquisition for a suitable selectivity in data analysis.

Quantification was carried out using external standard calibration curves in AFW matrix.

### 4.5. Method Validation

Method validation was accomplished with the evaluation of the selectivity, linearity, precision, sensibility, accuracy, ILOD, MLOD, and MLOQ using standard solutions of selected cyanotoxins and fortified AFW.

#### 4.5.1. Selectivity

For identification purposes, the exact mass of the precursor ion in the full-scan, the product ions in the fragmentation pattern, and the retention time of the standard in both solvent and spiked AFW were compared at a tolerance of ± 2.5%. Moreover, in accordance with the EURACHEM guidelines [45], the relative ion intensities of the product ions of the spiked samples were compared with the relative ion intensities obtained on the standard solutions, and at the same concentration levels as the ones used for the construction of the calibration curves.

#### 4.5.2. Linearity

The linearity of the measurements in the instrumentation was established by analyzing mixtures of the 10 targeted cyanotoxins at 16 different concentrations in the range of 1–50 µg/L. The Pearson’s correlation coefficient (R^2^) and the slope of the calibration curves in both solvent and AFW were determined. Two linear ranges were distinguished for each compound.

#### 4.5.3. Limits of Detection and Quantification

ILOD were experimentally determined by gradual dilutions of the standard solutions of selected cyanotoxins. The MLOD and MLOQ were based on matrix-matched calibration curve points. MLOD of each analyte was defined as the lowest concentration for which the peak area was, at least, three times the signal-to-noise, while the MLOQs were established as the lowest concentrations which fulfilled the criteria as a signal-to-noise ratio, at least, 10; relative standard deviation of three replicates, below 19%; Gaussian peak shapes; less than 5 ppm of exact mass error; and isotopic pattern similarity.

#### 4.5.4. Recoveries and Matrix Effects

The recovery was evaluated comparing responses of compounds in extracted samples with that of extracts of matrix spiked with standards post extraction. The recoveries were evaluated at three concentration levels (2, 10, and 20 ng/L for CYN, ANA, MC-RR, -YR, -LR, -LA, NOD; 1.5, 7.5, 15 ng/L for MC-LY, -LW, -LF). In all batch of samples, experimental blanks were analyzed.

The matrix effect was evaluated to determine a possible signal enhancement or ion suppression during the ionization process by interferents present in natural waters. To assess the matrix effects, fortified AFW and pure solvent were compared, and the percentage of effect was calculated according to the following expression:

% Matrix effects = ([Area]_AFW_ / [Area]_solvent_) × 100, being [Area]_AFW_ the integrated area of the cyanotoxin in the extracts and [Area]_solvent_ those corresponding to the pure solvent.

#### 4.5.5. Intra-Day and Inter-Day Precision

The inter-day precision was obtained as the average percentage of the relative standard deviation (RSD%) of standard solutions (six replicates) at seven concentration levels on three consecutive days. While, intra-day precision was determined using 10 replicate analysis of a standard solution at 1 µg/L, and expressed as the percentage of relative standard deviation (RSD).

#### 4.5.6. Accuracy

It was evaluated with the calculation of the recovery during the pre-treatment process. For this, fortified AFW were subjected to the pre-treatment process. Values obtained were compared with those from the extracts subjected to the same process but fortified after the sample treatment before the analysis.

## Figures and Tables

**Table 1:** Solid-phase extraction (SPE) optimization of elution conditions for Oasis HLB and Supelclean^TM^ ENVI-Carb^TM^ (± 1 standard deviation) in triplicate.

Compound	Conditions				
	10 mL MeOH	5 mL Heated MeOH	5 mL ACN	5 mL MeOH with 0.5% FA	5 mL MeOH with 0.1% NH_4_OH
**Oasis HLB**					
CYN	< 3	< 3	< 3	< 3	< 3
ANA	6.9 ± 1.9	10.2 ± 1.3	5.0 ± 0.4	20.2 ± 5.0	13.6 ± 3.6
MC-RR	55.0 ± 4.9	56.3 ± 0.7	5.5 ± 0.1	46.3 ± 2.2	37.9 ± 1.9
MC-YR	49.4 ± 3.6	46.4 ± 0.7	< 3	27.1 ± 0.9	35.6 ± 3.3
MC-LR	47.4 ± 4.0	44.3 ± 0.9	< 3	27.1 ± 0.6	33.2 ± 2.2
MC-LA	57.5 ± 3.7	58.5 ± 2.3	8.9 ± 0.4	32.8 ± 0.8	51.9 ± 2.1
MC-LW	13.6 ± 1.3	29.9 ± 10.2	< 1	< 1	36.3 ± 5.2
MC-LF	51.1 ± 3.5	63.1 ± 3.8	< 3	9.9 ± 2.0	63.6 ± 2.5
**Supelclean^TM^ ENVI-Carb^TM^**					
CYN	4.0 ± 0.6	< 3	< 3	22.6 ± 3.7	< 3
ANA	< 1	< 1	< 1	< 1	< 1
MC-RR	< 1	< 1	< 1	< 1	< 1
MC-YR	< 1	< 1	< 1	< 1	< 1
MC-LR	< 1	< 1	< 1	< 1	< 1
MC-LA	< 1	< 1	< 1	< 1	< 1
MC-LW	< 1	< 1	< 1	< 1	< 1
MC-LF	< 1	< 1	< 1	< 1	< 1

**Table 2:** Main analytical parameters.

Compound	Instrumental		Method						Precision, RSD%	
			Matrix Effect%	MLOD pg/L	MLOQ pg/L	Mean Recoveries, %				
	ILOD, pg	Linerity Range µg/L, R^2^				2 ng/L	10 ng/L	20 ng/L	Intraday	Interday
CYN	0.5	0.025–0.5, 0.9992	−59	100	300	53.4	52.2	87.2	5.2	2.0
		1–50, 0.9998								
ANA	0.2	0.01–0.25, 0.998	17	20	60	81.6	70.2	87.8	2.1	22.6
		0.5–50, 0.9998								
MC-RR	0.02	0.001–0.5, 0.9992	−11	4	12	72.2	62.8	66.6	1.6	17.9
		1–25, 0.9997								
NOD	0.5	0.025–0.25, 0.999	−35	100	300	81.1	66.1	82.1	1.5	17.3
		0.5–25, 0.9996								
MC-YR	1	0.05–0.1, 0.9928	−24	100	300	71.6	73.6	70.6	2.0	22.4
		0.25–50, 0.9943								
MC-LR	1	0.05–0.25, 0.998	−26	100	300	57.7	70.3	80.4	2.5	23.2
		0.5–50, 0.9992								
MC-LA	1	0.05–0.25, 0.9943	−23	100	300	82.8	70.0	80.0	2.7	17.7
		0.5–50, 0.9971								
MC-LY	0.76	0.038–0.75, 0.9995	15	75	225	84.3 ^a^	65.0 ^b^	80.6 ^c^	5.0	18.7
		2–38, 0.9993								
MC-LW	1.5	0.075–0.75, 0.9986	46	150	450	9.2 ^a^	32.3 ^b^	48.7 ^c^	8.8	14.1
		2–38, 0.9997								
MC-LF	0.76	0.038–0.75, 0.9994	35	75	225	63.9 ^a^	66.4 ^b^	70.2 ^c^	7.5	13.2
		2–38, 0.9994								

^a^ Concentration level 1.5 ng/L. ^b^ Concentration level 7.5 ng/L. ^c^ Concentration level 15 ng/L.

**Table 3:** Details on the optimized high-resolution mass spectrometry (HRMS) parameters for 10 targeted cyanotoxins.

Toxin	t_R_ (min)	Precursor Ion (*m/z*)	Product Ion (*m/z*)	CE (eV)
CYN	1.73	416.1241 [M + H]^+^	336.1664 [M + H − SO_3_] ^+^	30
ANA	1.75	166.1229 [M + H]^+^	149.0959 [M − NH_3_ + H]^+^	35
MC-RR	4.66	519.7902 [M + 2H]^2+^	135.0803 [C_9_H_11_O]^+^	30
NOD	4.89	825.4518 [M + H]^+^	135.0803 [C_9_H_11_O]^+^	32
MC-YR	4.97	1045.5353 [M + H]^1+^	135.0803 [C_9_H_11_O]^+^	30
MC-LR	5.03	995.5560 [M + H]^1+^	135.0803 [C_9_H_11_O]^+^	30
MC-LA	5.78	910.4904 [M + H]^+^	776.4176 [M + H − C_9_H_10_O]^+^	10
MC-LY	5.86	1002.5177 [M + H]^+^	868.4444 [M + H − C_9_H_10_O]^+^	10
MC-LW	6.23	1025.5334 [M + H]^+^	891.4594 [M + H − C_9_H_10_O]^+^	10
MC-LF	6.33	986.5253 [M + H]^+^	852.4490 [M + H − C_9_H_10_O]^+^	10

## Supplementary Materials

The following are available online at https://www.mdpi.com/2072-6651/12/4/247/s1. Table S1. Mean recoveries of Oasis HLB and SupelcleanTM ENVI-CarbTM at three concentration levels.; Table S2. Chromatographic parameters using different mobile phase compositions.; Table S3. Chromatographic parameters of LichtoCART® HPLC and Hibar® UHPLC columns.; Table S4. The most abundant m/z values for both positive and negative ionization modes.; Figure S1. Optimization of FA concentration in mobile phase: ACN (solvent A), H2O (solvent B) both with FA; Figure S2. Standard curves for targeted cyanotoxins.
