# Supplementary material for: Ultra-Trace Analysis of Cyanotoxins by Liquid Chromatography Coupled to High-Resolution Mass Spectrometry
Source: Toxins (Basel). 2020 Apr 11;12(4):247. doi: 10.3390/toxins12040247 (PMC7232229; doi:10.3390/toxins12040247)
Supplement: Supplementary file 1 [file toxins-12-00247-s001.pdf]

Supplementary Materials: Ultra-Trace Analysis of Cyanotoxins by Liquid Chromatography Coupled to High-Resolution Mass Spectrometry

Daria Filatova, Oscar Núñez and Marinella Farré

Table S1. Mean recoveries of Oasis HLB and Supelclean™ ENVI-Carb™ at three concentration levels.

Compound	Concentration, ng/L					
	Oasis HLB			Supelclean™ ENVI-Carb™		
	2	10	20	2	10	20
CYN	<3	<3	<3	53.4 ± 5.5	52.2 ± 2.1	87.2 ± 8.6
ANA	46.8 ± 6.7	25.6 ± 2.4	34.2 ± 1.9	34.8 ± 1.0	44.6 ± 1.8	53.0 ± 0.6
MC-RR	72.2 ± 7.3	62.8 ± 5.8	66.6 ± 7.5	<1	<1	<1
NOD	81.1 ± 5.1	66.1 ± 2.0	82.1 ± 2.4	<1	<1	<1
MC-YR	71.6 ± 9.0	73.6 ± 12.4	70.6 ± 6.7	<1	<1	<1
MC-LR	57.7 ± 9.7	70.3 ± 9.6	80.4 ± 8.5	<1	<1	<1
MC-LA	82.8 ± 4.8	70.0 ± 7.1	80.0 ± 2.8	<1	<1	<1
MC-LY	84.3 ± 4.9 [a]	65.0 ± 5.7 [b]	80.6 ± 5.9 [c]	<1	<1	<1
MC-LW	9.2 ± 2.2 [a]	32.3 ± 4.1 [b]	48.7 ± 7.2 [c]	<1	<1	<1
MC-LF	63.9 ± 7.1 [a]	66.4 ± 12.0 [b]	70.2 ± 4.7 [c]	<1	<1	<1

[a] Concentration level 1.5 ng/L. [b] Concentration level 7.5 ng/L. [c] Concentration level 15 ng/L.

Table S2. Chromatographic parameters using different mobile phase compositions.

Compound	Solvent A with 0.1% of FA	Retention Time, tR, min	Retention Factor, K	Tailing Factor, Tf	Selectivity, $\alpha_{(a,a+1)}$	Resolution, Rs	Peak Width at the Base, Wb
CYN	ACN	1.68	0.39	0.73	0.98	0.03	0.18
	MeOH	1.73	0.40	0.7	1.02	0.03	0.18
ANA	ACN	1.67	0.38	1.14	13.76	13.81	0.5
	MeOH	1.74	0.40	2.13	17.78	19.74	0.47
MC-RR	ACN	7.54	5.23	1.28	1.06	1.21	0.35
	MeOH	10.13	7.17	1.43	1.06	1.51	0.38
NOD	ACN	7.92	5.55	1.24	1.03	0.75	0.28
	MeOH	10.68	7.61	1.08	1.00	0.06	0.35
MC-YR	ACN	8.1	5.69	1.31	1.01	0.21	0.2
	MeOH	10.7	7.63	0.93	1.02	0.48	0.29
MC-LR	ACN	8.15	5.74	1.47	1.31	9.06	0.27
	MeOH	10.85	7.75	1.3	1.14	5.00	0.33
MC-LA	ACN	10.28	7.50	0.7	1.01	0.37	0.2
	MeOH	12.15	8.80	0.7	1.00	0.15	0.19
MC-LY	ACN	10.36	7.56	0.88	1.10	3.36	0.23
	MeOH	12.12	8.77	0.95	1.03	1.65	0.2
MC-LW	ACN	11.3	8.34	0.83	1.04	1.08	0.33
	MeOH	12.45	9.04	1.22	1.02	0.93	0.2
MC-LF	ACN	11.75	8.71	1.08			0.5
	MeOH	12.65	9.20	1.66			0.23

Conditions for chromatographic separation: gradient elution was performed with 0.1% formic acid acetonitrile (solvent A) and 0.1% formic acid aqueous solution (solvent B) at a constant flow rate of 0.2 mL min^{-1} with the following gradient program: 0–3 min at 10% A; 3–11 min from 10 to 90% A; 11–16 min at 90%

B; 16–18 min back to the initial conditions at 10% A; and column re-equilibration for 7 min under the initial conditions. The injection volume was 20 μL.

Table S3. Chromatographic parameters of LichtoCART® HPLC and Hibar® UHPLC columns.

Compound	Column	Retention Time, tR, min	Retention Factor, K	Tailing Factor, Tf	Selectivity, $\alpha_{(a,a+1)}$	Resolution, Rs	Peak Width at the Base, Wb
CYN	HPLC	1.68	0.39	0.73	0.98	0.03	0.18
	UHPLC	1.69	0.72	1.04	1.25	1.89	0.10
ANA	HPLC	1.67	0.38	1.14	13.76	13.81	0.5
	UHPLC	1.87	0.91	1.13	3.34	18.09	0.09
MC-RR	HPLC	7.54	5.23	1.28	1.06	1.21	0.35
	UHPLC	3.95	3.03	1.1	1.08	2.00	0.14
NOD	HPLC	7.92	5.55	1.24	1.03	0.75	0.28
	UHPLC	4.18	3.27	1.05	1.02	0.74	0.09
MC-YR	HPLC	8.1	5.69	1.31	1.01	0.21	0.2
	UHPLC	4.25	3.34	1.04	1.02	0.51	0.10
MC-LR	HPLC	8.15	5.74	1.47	1.31	9.06	0.27
	UHPLC	4.3	3.39	1.1	1.20	5.00	0.10
MC-LA	HPLC	10.28	7.50	0.7	1.01	0.37	0.2
	UHPLC	4.97	4.07	1.02	1.02	0.41	0.17
MC-LY	HPLC	10.36	7.56	0.88	1.10	3.36	0.23
	UHPLC	5.03	4.13	1.1	1.08	2.14	0.12
MC-LW	HPLC	11.3	8.34	0.83	1.04	1.08	0.33
	UHPLC	5.34	4.45	1.1	1.02	0.57	0.17
MC-LF	HPLC	11.75	8.71	1.08			0.5
	UHPLC	5.44	4.55	1.11			0.18

Conditions for chromatographic separation for UHPLC coulunm: gradient elution was performed with 0.05% formic acid acetonitrile (solvent A) and 0.05% formic acid aqueous solution (solvent B) at a constant flow rate of 0.3 mL min^{-1} with the following gradient program: 0–1 min at 10% A; 1–5min from 10 to 90% A; 5–8 min at 90% B; 8–8.5 min back to the initial conditions at 10% A; and column re-equilibration for 1.5 min under the initial conditions. The injection volume was 20 μL.

Table S4. The most abundant *m*/*z* values for both positive and negative ionization modes.

Compound	Elemental Composition	Neutral Mass, Theoretical	Ion Specie in Positive Mode	Theoretical *m*/*z* in Positive Mode	Mass Error in Positive Mode (ppm)	Ion Specie in Negative Mode	Theoretical *m*/*z* in Negative Mode	Mass Error in Negative Mode (ppm)
CYN	$C_{15}H_{21}N_5O_7S$	415.1156	$[M+H]^+$	416.1234	−0.48	$[M-H]^-$	414.1089	−2.17
ANA	$C_{10}H_{15}NO$	165.1148	$[M+H]^+$	166.1226	1.81	$[M-H]^-$	164.1081	N/D
MC-RR	$C_{49}H_{75}N_{13}O_{12}$	1037.5664<br>518.7824	$[M+2H]^{2+}$	519.7902	0.96	$[M-H]^-$	1036.5585	0.09
NOD	$C_{41}H_{60}N_8O_{10}$	824.4438	$[M+H]^+$	825.4505	1.57	$[M-H]^-$	823.4360	−1.21
MC-YR	$C_{52}H_{72}N_{10}O_{13}$	1044.5286<br>522.2635	$[M+H]^{2+}$	523.2713	1.72	$[M-2H]^{2-}$	521.2567	−1.73
MC-LR	$C_{49}H_{74}N_{10}O_{12}$	994.5488<br>497.2738	$[M+H]2^+$	498.2817	1.61	$[M-H]^-$	993.5415	−0.80
MC-LA	$C_{46}H_{67}N_7O_{12}$	909.4848	$[M+H]^+$	910.4920	1.31	$[M-H]^-$	908.4775	−1.32
MC-LY	$C_{52}H_{71}N_7O_{13}$	1001.5110	$[M+H]^+$	1002.5183	−0.59	$[M-H]^-$	1000.5037	−1.09
MC-LW	$C_{54}H_{72}N_8O_{12}$	1024.5270	$[M+H]^+$	1025.5342	1.56	$[M-H]^-$	1023.5197	−1.07
MC-LF	$C_{52}H_{71}N_7O_{12}$	985.5233<br>492.7575	$[M+H]^+$	986.5223	0.71	$[M-2H]^{2-}$	491.7508	−2.03

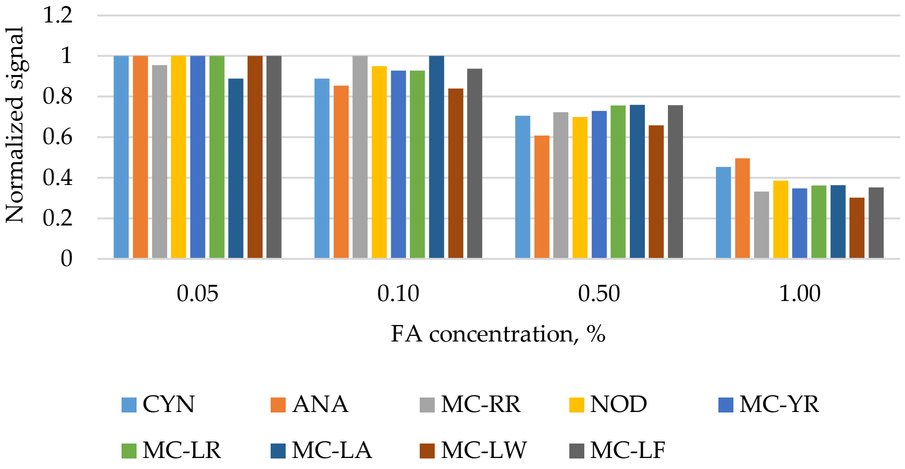

Figure S1. Optimization of FA concentration in mobile phase: ACN (solvent A), H_2O (solvent B) both with FA.

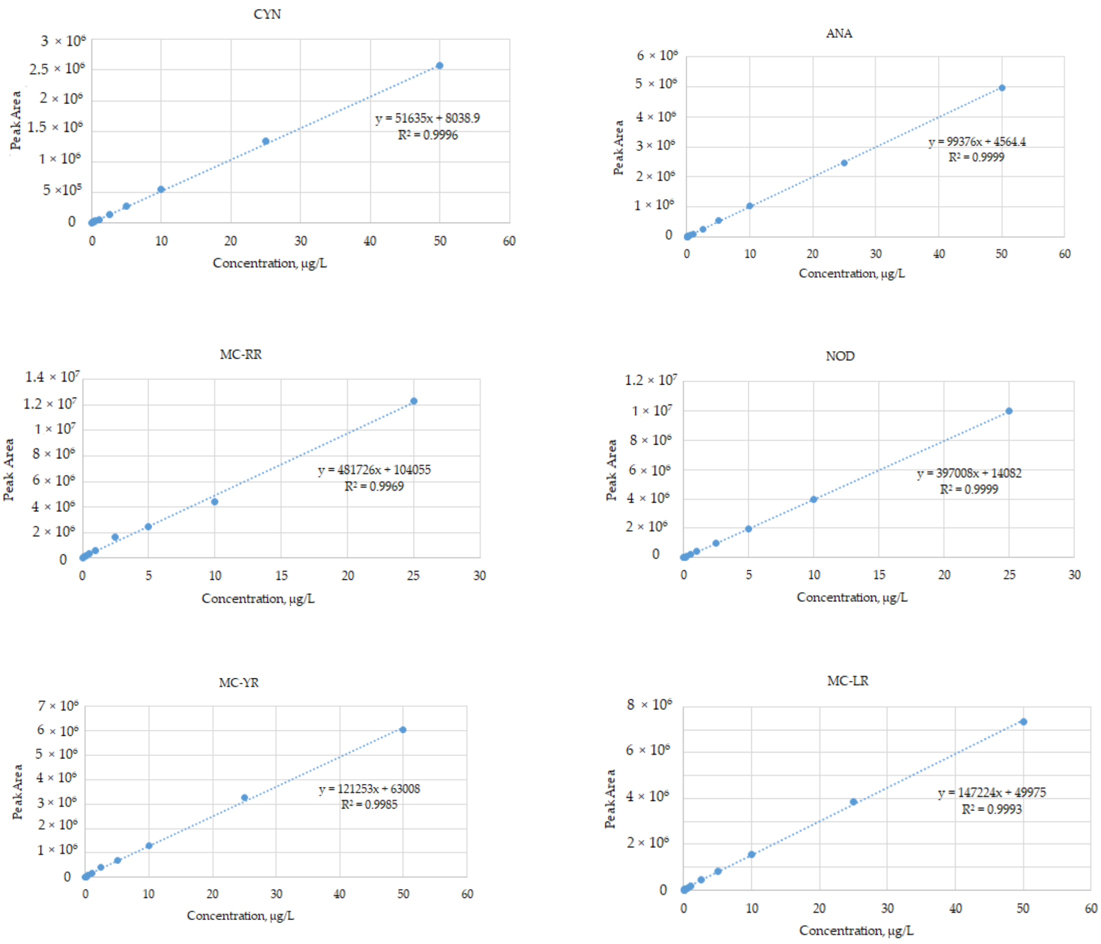

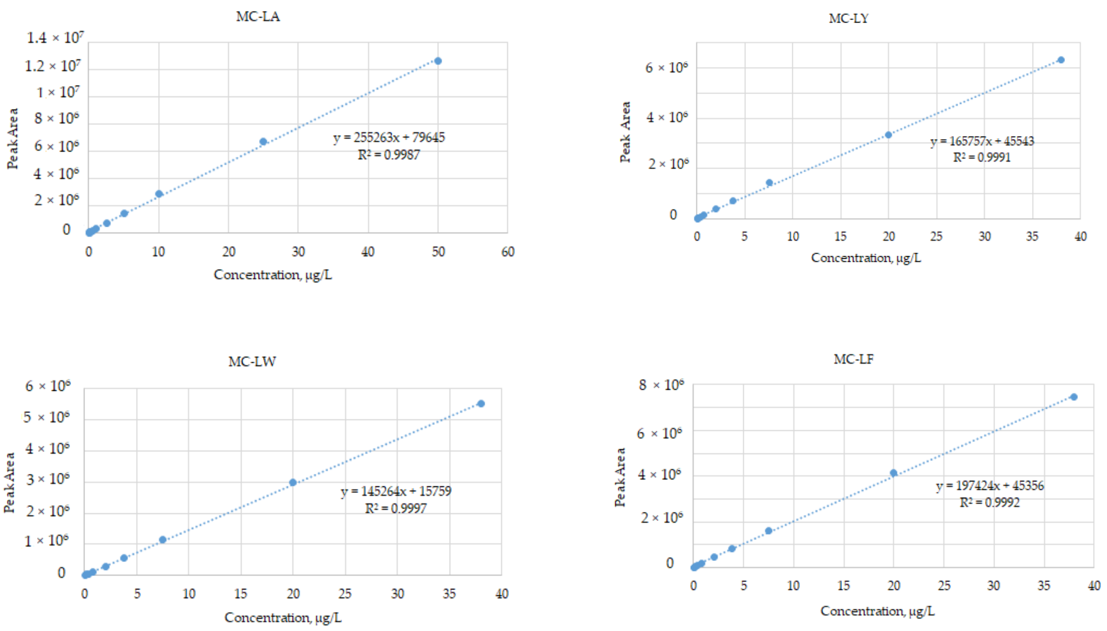

Figure S2. Standard curves for targeted cyanotoxins.
